# Addition of dexmedetomidine to benzodiazepines for patients with alcohol withdrawal syndrome in the intensive care unit: a randomized controlled study

**DOI:** 10.1186/s13613-015-0075-7

**Published:** 2015-11-02

**Authors:** Kateryna Bielka, Iurii Kuchyn, Felix Glumcher

**Affiliations:** Department of Anesthesiology and Intensive Care, Bogomolets National Medical University, 13 T. Shevchenko Boulevard, 01601 Kiev, Ukraine

**Keywords:** Alcohol withdrawal syndrome, Dexmedetomidine, Benzodiazepines, Sedation, Randomized controlled trial

## Abstract

**Background:**

Dexmedetomidine (DEX) is a centrally acting alpha-2-adrenoceptor agonist that has potential in the management of alcohol withdrawal syndrome (AWS) owing to its ability to produce arousable sedation and to inhibit the adrenergic system without respiratory depression. The objective of this randomized controlled study was to evaluate whether addition of DEX to benzodiazepine (BZD) therapy is effective and safe for AWS patients in the intensive care unit (ICU).

**Methods:**

Eligible participants were randomly assigned to intervention (Group D; *n* = 36) or control (Group C; *n* = 36). In Group D, DEX infusion was started at a dose of 0.2–1.4 μg/kg/h and titrated to achieve the target sedation level (–2 to 0 on the Richmond Agitation Sedation Scale (RASS)) with symptom-triggered BZD (10 mg diazepam bolus) was used as needed. Patients in Group C received only symptom-triggered 10 mg boluses of diazepam. The primary efficacy outcomes were 24-h diazepam consumption and cumulative diazepam dose required over the course of the ICU stay; secondary outcomes included length of ICU stay, sedation and communication quality and haloperidol requirements.

**Results:**

Median 24-h diazepam consumption during the study was significantly lower in Group D (20 vs. 40 mg, *p* < 0.001), as well as median cumulative diazepam dose during the ICU stay (60 vs. 90 mg, *p* < 0.001). The median percentage of time in the target sedation range was higher in Group D (median 90 % (90–95) vs. 64.5 % (60–72.5; *p* < 0.001). DEX infusion was also associated with better nurse-assessed patient communication (<0.001) and fewer patients requiring haloperidol treatment (2 vs. 10 *p* = 0.02). One patient in Group D and four in Group C were excluded owing to insufficient control of AWS symptoms and use of additional sedatives (*p* = 0.36). There were no severe adverse events in either group. Spontaneous breathing remained normal in all patients. Bradycardia was a common adverse event in Group D (10 vs. 2; *p* = 0.03).

**Conclusions:**

DEX significantly reduced diazepam requirements in ICU patients with AWS and decreased the number of patients who required haloperidol for severe agitation and hallucinations. DEX use was also associated with improvement in diverse aspects of sedation quality and the quality of patient communication.

Trial registration: ClinicalTrials.gov: NCT02496650

## Background

Alcohol withdrawal syndrome (AWS) often complicates the perioperative period or critical illnesses and may increase the likelihood of admission to the intensive care unit (ICU). Up to 20 % of hospitalized patients have alcohol dependence and 18 % of these patients will develop AWS during their hospital stay [1]. The symptoms of AWS usually emerge 24–96 h after cessation of alcohol consumption and are characterized by sympathetic hyperactivity and metabolic and psychiatric disorders (e.g. agitation, hallucination and seizures). Benzodiazepines (BZDs) are commonly used to manage AWS and are effective for that purpose. Several studies have shown that BZDs reduce the incidences of seizures and delirium and shorten the duration of AWS compared with placebo [2, 3]. However, BZD monotherapy may not be sufficient to control AWS symptoms [1], and administration of large doses is associated with excessive sedation, respiratory failure, worsening of delirium, increased aspiration and intubation risks, increased length of hospitalization and increased hospital costs [3, 4]. Furthermore, chronic liver disease patients are at risk of oversedation and progression of hepatic encephalopathy while using BZDs [5].

Alternative drugs with good efficacy and safety profiles are currently unavailable for the management of AWS [6]. Anticonvulsants, antipsychotics, ethanol, barbiturates and propofol have historically been used for this purpose [7, 8], but the evidence base for these agents is weak or absent [6]. There have also been several studies of clonidine as adjunct treatment for AWS in the ICU. Significant decreases in AWS symptoms [9] and BZD doses [10] were reported, but with greater risk of adverse events such as bradycardia and hypotension [10, 11]. Disadvantages of clonidine include that it produces only a mild sedative effect (but with a significant hemodynamic impact) and its long duration of action (up to 12–16 h) [7].

Dexmedetomidine (DEX) is a selective, central ɑ_2_-receptor agonist which is approved for ICU sedation in mechanically ventilated patients and for procedural sedation for non-intubated patients [12]. Compared with clonidine, DEX offers more effective sedative and analgesic properties, a shorter half-life (2–3 h) and significantly lower rates of hemodynamic complication [12, 13]. Moreover, DEX does not cause respiratory depression and decreases the duration of mechanical ventilation [12, 13].

There have been various reports of the successful use of DEX—usually as an adjunct to BZDs—in the management of AWS during the last 10 years. However, most publications have been limited to case reports [14], case series [15] and retrospective [1] and prospective observational studies [16]. Only one randomized controlled study has been published to date [17], in which the authors found that adjunct use of DEX was associated with DEX attenuation of AWS symptoms, with concomitant reductions in use of BZDs. The commonest side effects were hypotension and bradycardia.

The objective of this randomized controlled study was to evaluate whether addition of DEX to BZD therapy is effective and safe for AWS patients in the ICU. We hypothesized that DEX would reduce BZD consumption and the need for neuroleptics, as well as improving sedation quality.

## Methods

This randomized, single-center, controlled study was conducted in the adult mixed ICU at the private hospital ‘Boris’ in Kiev, Ukraine. The inclusion criteria were: age ≥18–75 years and AWS diagnosed by means of the Diagnostic and Statistical Manual of Mental Disorders, 4th Edition criteria [18], plus the signed informed consent of either the patient or the patient’s family or a legal representative. The exclusion criteria were age outside the specified range, history of use of other psychoactive substances or of withdrawal states, general anesthesia during the previous 24 h or other sedatives used, severe neurologic disorder (traumatic brain injury, acute stroke, severe dementia), pregnancy or lactation, severe comorbidities (severe heart failure, acute myocardial infarction, heart rate <50 beats/min, glomerular filtration rate <30 mL/min, liver failure Child-Pugh class C, acute respiratory failure) and known allergy to the study medication.

Typical reasons for ICU admission were severe agitation, hallucinations, Clinical Institute Withdrawal Assessment of Alcohol Scale, Revised (CIWA-Ar) score ≥15, history of seizures or previous delirium tremens (DT), coexisting medical problems (e.g. pancreatitis) or respiratory distress.

After the primary patient assessment, eligible participants were assigned in a 1:1 ratio to either the intervention (Group D) or control (Group C) groups using random assignment in blocks of four. The randomization sequence was generated using a computer algorithm [19]. Randomization and data analysis were conducted by an independent blinded member of the research team.

In Group D, DEX infusion was started at a dose of 0.2–1.4 μg/kg/h and titrated to achieve the target sedation level of –2 to 0 on the Richmond Agitation Sedation Scale (RASS) and CIWA-Ar score <15. DEX loading doses were not used. Dosing and duration of DEX infusion were adjusted by the clinical management team based on sedation assessment (with duration of DEX infusion no longer than 72 h). In patients for whom increasing the DEX infusion rate to 1.4 μg/kg/h did not achieve RASS −2 to 0 and/or a CIWA-Ar score of <15, diazepam (10 mg i.v.) was administered according to a symptom-triggered protocol. In Group C, the same symptom-triggered diazepam regimen protocol was used. In both groups diazepam was administered every 30 min as needed to control active withdrawal symptoms (CIWA-Ar score ≥15 or RASS score >+2), as prescribed by the clinical management team. Antipsychotics (i.m. haloperidol, 5 mg boluses) were used as a rescue medication in both groups for severe agitation or hallucinations. Haloperidol was prescribed only if the QT interval (QT_c_) was documented to be normal. No other sedatives or analgesics were allowed during the study period.

The primary efficacy outcomes were median 24-h diazepam consumption and median cumulative diazepam dose required over the course of the ICU stay.

Secondary efficacy outcomes included:

- length of ICU stay and intubation rates;
- sedation quality: time in the target sedation range [RASS score 0 to −2] as a proportion of total sedation time; the duration of insufficient sedation: time with RASS score ≥+2 as a proportion of total sedation time; the duration of oversedation : time with RASS score ≤–3] expressed as a proportion of total sedation time; and the number of rescue sedation boluses and sedation stops required over a 24-h period;
- the ability to communicate, such as asking for help or answering questions on comfort and pain, which was assessed by nurses during each shift (every 12 h) on a scale from 0 to 10, where 0 = uncommunicative and 10 = patient communicates well;
- haloperidol requirements (number of patients who received haloperidol for severe agitation and hallucinations) and cumulative haloperidol dose.

During their ICU stay, patients in both groups were evaluated by the nursing staff using the RASS and the CIWA-Ar scale (either every 2 h or prior to rescue therapy). The level of alertness was assessed using the Observer’s Assessment of Alertness/Sedation (OAA/S) scale every 2 h.

Safety was assessed by monitoring vital signs, performing laboratory tests (partial oxygen pressure in arterial blood [PaO_2_], partial carbon dioxide pressure in arterial blood [PaCO_2_], oxygen saturation in arterial blood [SaO_2_], blood glucose) and recording adverse events. Continuous invasive measurement of blood pressure and pulse and continuous electrocardiogram monitoring was performed for all patients. QT_c_ monitoring was provided for patients treated with haloperidol. Spontaneous breathing was assessed using continuous respiratory rate monitoring and pulse oximetry. Arterial blood gases were checked every 12 h or less as determined by the attending clinician.

An adverse event was recorded if systolic blood pressure was <90 or >160 mmHg or if heart rate was <50 or >110 beats/min; desaturation was estimated as peripheral oxygen saturation (or SaO_2_) <90 %; hypoglycemia was defined as serum glucose <3.9 mmol/L and hyperglycemia as serum glucose >10 mmol/L. Interventions for bradycardia, tachycardia, hypertension and hypotension comprised titration or interruption of study agent, or additional drug therapy.

Statistical analysis was performed using Statistica 8.0 and R software (StatSoft Inc., Tulsa, OK, USA). Categorical data are presented as proportions and continuous data as medians with 25–75 % interquartile ranges (IQRs). Chi square testing demonstrated that all of the study variables were discrete. To assess significance levels, a two-tailed Mann–Whitney *U* test and Fisher’s exact test were used. A *p* value of <0.05 was considered significant.

This study was approved by the Bogomolets National Medical University ethics committee (approval code number 84).

## Results and discussion

A total of 72 patients were randomized to the study groups (*n* = 36 per group). The median time from hospital admission to the start of the study was 24 h (IQR 12–48 h) in Group D and 30 h (IQR 20–50 h) in Group C (*p* = 0.9). There were no significant differences between the study groups regarding demographic characteristics, comorbidities, initial AWS severity and diazepam dose administered prior to study enrollment.

Baseline characteristics of the study population are presented in Table 1.

**Table 1:** Demographic data and AWS severity at baseline

	Group D (DEX)	Group C (control)	*p* value
Male	33/35 (94)	28/32 (88)	0.9
Age, median (IQR)	46.5 (43–50)	46 (42–50)	1.0
Comorbidity			
Liver cirrhosis			
Child-Pugh A, n (%)	1/35 (3)	1/32 (3)	1.0
Child-Pugh B, n (%)	2/35 (6)	1/32 (3)	0.9
Pneumonia, n (%)	1/35 (3)	0/32	1.0
Diabetes, n (%)	1/35 (3)	2/32 (6)	0.9
Congestive heart failure			
NYHA class I	1/35 (3)	1/32 (3)	1.0
NYHA class II	1/35 (3)	1/32 (3)	1.0
Arterial hypertension n (%)	3/35 (9)	6/32 (19)	0.19
Other			
Leg fracture, n (%)	2/35 (6)	0/32	1.0
Acute pancreatitis, n (%)	1/35 (3)	2/32 (6)	1.0
CIWA-Ar at ICU admission, median (IQR)	25 (18 to 29)	26 (17–28)	1.0
RASS at ICU admission, median (IQR)	+2 (+1 to +3)	+2 (+1 to +3)	1.0
Diazepam dose administered prior to study enrollment, mg, median (IQR)	30 (20–40)	30 (20–40)	1.0

*CIWA-Ar* Clinical Institute Withdrawal Assessment of Alcohol Scale, Revised, *IQR* interquartile range, *NYHA* Ne``w York Heart Association Functional Classification

**Fig. 1:**
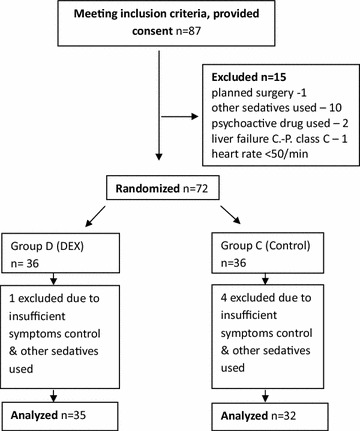
CONSORT flowchart

The median duration of DEX infusion was 36 h (IQR 24–42 h) with a median dose of 0.5 μg/kg/h (IQR 0.4–0.8 μg/kg/h). All patients survived to discharge (Fig. 1).

**Fig. 2:**
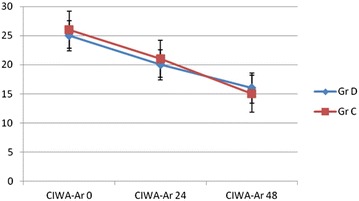
Diazepam 24-h consumption and cumulative dose during ICU stay

The main outcomes of the study are presented in Table 2 and Fig. 2. As shown therein all the pre-specified dimensions of AWS symptomatology, BZD consumption, sedation quality, patient alertness and ability to communicate and use of rescue medications were favorably influenced by the use of DEX.

**Table 2:** Efficacy outcomes in study groups

	Group D (DEX)	Group C (control)	*p* value
Diazepam consumption in 24 h, mg	20 (20–30)	40 (40–50)	<0.001
Cumulative diazepam consumption, mg	60 (50–60)	90 (80–100)	<0.001
Time of target sedation, %	90 (90–95)	64.5 (60–72.5)	<0.001
Time of insufficient sedation, %	7.75 (5–10)	15 (10–20)	<0.001
Time of oversedation, %	2 (0–5)	15 (10–20)	<0.001
Rescue sedation boluses, no. in 24 h	1.25 (0–4)	4 (3–6)	0.004
Sedation stops, no. in 24 h	0 (0–1)	2 (0–3)	0.001
Communication with patient	9 (7–10)	6 (5–6)	<0.001
OAA/S scale	1 (0–2)	2 (1–4)	0.03
Haloperidol use, no. of patients (%)	2/32 (6)	10/32 (31)	0.02
Median cumulative haloperidol dose, mg	50 (IQR 40–55)	60 (IQR 40–65)	0.2

Value expressed as medians (Interquartile Ranges 25 to 75), unless otherwise specified

*No* number, *OAA/S* Observer’s Assessment of Alertness/Sedation

In this randomized controlled study, addition of DEX to BZD therapy significantly decreased 24-h diazepam consumption and cumulative diazepam dose during the ICU stay in AWS patients.

Sedation quality, one of the secondary outcomes, differed significantly between the two groups across a range of outcome indices. The median time in the target sedation range was significantly higher in Group D (Δ25 %; *p* < 0.001) and DEX infusion was associated with better patient communication (Δ3 points; *p* < 0.001). The duration of excessive sedation, number of sedation stops, duration of insufficient sedation and number of rescue sedation boluses were all significantly higher in Group C (Table 2). CIWA-Ar psychometric values decreased in both groups with the course of AWS, with no significant differences in median values between the two groups.

The number of patients who received haloperidol as a rescue medication for severe agitation and hallucinations was lower in Group D (odds ratio 6.8, 95 % confidence interval 1.4–33). However, the cumulative dose of haloperidol during the ICU stay did not differ significantly between groups: median cumulative dose 50 mg (IQR 40–55 mg) in Group D and 60 mg (IQR 40–65 mg) in Group C (*p* = 0.2). Other authors have reported significant reduction in haloperidol use after addition of DEX [1, 20], although those studies were retrospective and had several design limitations. In the only controlled trial of DEX as adjunctive therapy for AWS reported to date, haloperidol consumption and doses were not studied [17].

The median length of ICU stay was 50 h (IQR 46–65 h) in Group D and 70 h (IQR 65–90 h) in Group C (*p* = 0.059). DT developed in one patient in Group D and four in Group C (*p* = 0.36). These patients were excluded from the analysis due owing to insufficient control of AWS symptoms with study medication (e.g. dexmedetomidine, diazepam, haloperidol) and other sedatives used (propofol), all of them were intubated for airway protection and respiratory support. To our knowledge, there is no robust evidence of benefit from DEX use in patients with DTs. One prospective trial reported benefits with addition of DEX to BZD therapy in patients with DT [16]. However, that study had significant limitations: there was no comparison or control group and delirium was defined using the Confusion Assessment Method for the Intensive Care Unit (CAM-ICU), with no clear distinction between delirium caused by AWS and other factors.

Spontaneous breathing remained normal during the study period in all patients; desaturation was successfully treated with administration of extra oxygen and sedative drug titration. There were no statistically significant differences between Groups D and C regarding desaturation incidence (Table 3), respiratory rate and arterial PaO_2_ or PaCO_2_.

**Table 3:** Complications and adverse events rates in both groups

	Group D (DEX)	Group C (control)	Odds ratio (CI 95 %)	*p* value
Adverse events				
Hypotension, *n* (%)	8/35 (23)	4/32 (13)	2 (0.48–11)	0.34
Hypertension, *n* (%)	0/35	4/32 (16)	11 (0.6–190)	0.05
Tachycardia, *n* (%)	0/35	5/32 (16)	14 (0.9–283)	0.02
Bradycardia, *n* (%)	10/35 (31)	2/32 (6)	6 (1.3–73)	0.03
Desaturation, *n* (%)	1/35 (3)	5/32 (15)	5.3 (0.6–280)	0.2
Hypoglycemia, *n* (%)	2/35 (6)	1/32 (3)	2 (0–114)	1.0
Hyperglycemia, *n* (%)	5/35 (14)	9/32 (28)	2 (0.7–9)	0.2
Complications				
Hospital pneumonia, *n* (%)	1/35 (3)	2/32 (6)	2 (0.1–125)	0.6

No severe adverse events were identified (Table 3). The commonest adverse events observed were hypotension, hypertension and desaturation, with similar incidence in study groups; bradycardia, which was observed significantly more often in Group D (*p* = 0.01); and tachycardia, which was observed significantly more often in Group C (*p* = 0.02). Hospital-acquired pneumonia was diagnosed in one patient in Group D and two in Group C. No seizures were observed during the study.

After ICU discharge, patients continued treatment in the general department ward with oral diazepam. The median duration of hospitalization was 9 days (IQR 8–10 days) in Group D and 11 days (IQR 10–13 days) in Group C (*p* = 0.034).

DEX is an attractive drug for AWS management owing to its ability to produce arousable sedation and to inhibit the adrenergic system without respiratory depression [12]. The benefits of DEX in AWS management have been shown in several retrospective series [1, 13–15]. Rayner et al. [1] published a retrospective review of 20 AWS patients admitted to the ICU, with DEX being used in addition to BZDs. The mean dose of DEX was 0.53 µg/kg/h and the mean duration of therapy was 49.1 h. Adjunctive DEX decreased severity score, haloperidol use and diazepam dose within 4 h. Dailey et al. [21] published a retrospective chart review of 10 patients with AWS who were treated with DEX. The mean dose was 0.7 µg/kg/h and the mean infusion time was 50 h. The authors reported a significant diazepam dose reduction from 13 mg/h prior to DEX infusion to 3 mg/h in the 24 h after treatment. All patients in the study had normal spontaneous breathing. Other studies have reported similar results, although the majority of them were observational or retrospective [14–16].

To date only a few prospective controlled studies of use of DEX in AWS patients have been published [16, 17]. The authors of that research concluded that DEX shows promise as an adjunct to BZDs but concluded that further studies are needed to fully profile the clinical impact of DEX in AWS. Our own study is a modest addition to the dataset of prospectively derived data and is supportive of earlier conclusions but it is still necessary to conduct larger randomized trials of DEX in AWS.

The limitations of this study include the partially blinded design with absence of placebo control and the small sample size (*n* = 72), which make it difficult to draw definitive conclusions. The exclusion of all patients who developed DT precludes any conclusions of the effectiveness of DEX in that situation but the indications are that it adds little to the treatment options for that aspect of AWS.

Nevertheless, this trial supports adjunctive DEX use for many AWS patients in the ICU and provides efficacy and safety outcomes. In the authors’ opinion, we now have enough data to consider DEX as a valid adjunct to BZD therapy for AWS patients in the ICU. However, we need more data and new large studies before definite medical conclusions can be reached and the question of cost-effectiveness has still to be addressed.

## Conclusions

Addition of DEX to BZD therapy is effective and safe for AWS patients in the ICU. DEX appears to significantly reduce diazepam requirements as well as improve both sedation quality and patient communication. Addition of DEX to diazepam decreased the number of patients who required haloperidol for severe agitation. Monitoring for bradycardia is necessary during DEX infusion.

## Abbreviations

AWS: alcohol withdrawal syndrome
BZD: benzodiazepine
CAM-ICU: Confusion Assessment Method for the Intensive Care Unit
CIWA-Ar: Clinical Institute Withdrawal Assessment of Alcohol Scale, Revised
DEX: dexmedetomidine
DT: delirium tremens
ICU: intensive care unit
IQR: interquartile range
OAA/S: Observer’s Assessment of Alertness/Sedation
PaCO_2_: partial carbon dioxide pressure in arterial blood
PaO_2_: partial oxygen pressure in arterial blood
QT_c_: corrected QT interval
RASS: Richmond Agitation Sedation Scale
SaO_2_: oxygen saturation in arterial blood
SpO_2_: peripheral oxygen saturation
